# Correction: Harnessing the power of AI: Advanced deep learning models optimization for accurate SARS-CoV-2 forecasting

**DOI:** 10.1371/journal.pone.0296111

**Published:** 2023-12-14

**Authors:** Muhammad Usman Tariq, Shuhaida Binti Ismail, Muhammad Babar, Ashir Ahmad

The Acknowledgements section is missing in the paper. The correct Acknowledgements is as follows: The authors would like to acknowledge the support of Prince Sultan University for paying the Article Processing Charges (APC) of this publication.
